# An Artificial Bee Colony Algorithm for Uncertain Portfolio Selection

**DOI:** 10.1155/2014/578182

**Published:** 2014-06-26

**Authors:** Wei Chen

**Affiliations:** School of Information, Capital University of Economics and Business, Beijing 100070, China

## Abstract

Portfolio selection is an important issue for researchers and practitioners. In this paper, under the assumption that security returns are given by experts' evaluations rather than historical data, we discuss the portfolio adjusting problem which takes transaction costs and diversification degree of portfolio into consideration. Uncertain variables are employed to describe the security returns. In the proposed mean-variance-entropy model, the uncertain mean value of the return is used to measure investment return, the uncertain variance of the return is used to measure investment risk, and the entropy is used to measure diversification degree of portfolio. In order to solve the proposed model, a modified artificial bee colony (ABC) algorithm is designed. Finally, a numerical example is given to illustrate the modelling idea and the effectiveness of the proposed algorithm.

## 1. Introduction

Portfolio selection deals with the problem of how to allocate investor's wealth among different assets such that the investment goal can be achieved. Markowitz [1] originally proposed the mean-variance (M-V) model for portfolio selection in 1952, which has played an important role in the development of modern portfolio selection theory. The key principle of the M-V model is to use the expected return of a portfolio as the investment return and to use the variance (or standard deviation) of the expected returns of the portfolio as the investment risk. Markowitz's M-V model for portfolio selection problems can be formulated mathematically in two ways: minimizing risk under prescribing a minimum acceptable expected return level and maximizing expected return under prescribing a maximum acceptable risk level. Since then, a number of scholars, including Sharp [2], Merton [3], Pang [4], Perold [5], Best and Grauer [6], Konno and Yamazaki [7], and Best and Hlouskova [8], proposed different mathematical methods for the development of portfolio models. All the above-mentioned researches assume that the security returns are random variables with probability distributions. In probability theory, probability density and probability distribution functions of a random variable are usually derived from historical data. However, in the real securities market, market imperfections make the security returns present vagueness and ambiguity so that sometimes security returns cannot be well reflected by historical data. Therefore, the prediction of security returns is determined largely by experts' estimation and contains much subjective imprecision rather than randomness. With the extensive application of fuzzy set theory [9], many researchers, such as Tanaka and Guo [10], Carlsson et al. [11], Vercher et al. [12], Zhang et al. [13], Chen et al. [14], Tsaur [15], and Gupta et al. [16], have investigated the portfolio selection problem in fuzzy environment.

An important assumption for the above fuzzy portfolio problems is that security returns are fuzzy variables. However, as we research the problem deeper, we find that paradoxes will appear if fuzzy set is employed. For example, if a security return is regarded as a fuzzy variable, then we may assign it a membership function, suppose it is a triangular fuzzy variable *ξ* = (−0.02,0.03,0.08). Based on the membership function, the possibility theory will immediately conclude the following three propositions: (a) the return is exactly 0.03 with possibility measure 1; (b) the return is not 0.03 with possibility measure 1; and (c) “return is exactly 0.03” and “return is not exactly 0.03” are equally likely. However, it is doubtless that the belief degree of “return is exactly 0.03” is almost zero. On the other hand, “return is exactly 0.03” and “return is not exactly 0.03” have the same belief degree in possibility measure, which implies that the two events will happen equally likely. It seems that no human being can accept this conclusion. This paradox shows that those imprecise quantities like security returns cannot be quantified by possibility measure and then they are not fuzzy concepts. To avoid paradox, Liu [17] founded the uncertainty theory based on an axiomatic system of normality, self-duality, countable subadditivity, and product uncertain measure. Based on the uncertainty theory, much work has been done on the development of theoretical and practical work. Peng and Iwamura [18] proved a sufficient and necessary condition of uncertainty distribution. Chen and Liu [19] proved the existence and uniqueness theorem for uncertain differential equations. Gao [20] investigated the *α*-shortest path and the shortest path problem in uncertain networks. Ding and Gao [21] assumed that demand is uncertain variable and formulated an optimal (*σ*, *S*) policy for uncertain multiproduct newsboy problem. In the area of uncertain portfolio selection problems, Zhu [22] solved an uncertain optimal control problem and applied it to a portfolio selection model. Taking semiabsolute deviation as a risk measure, Liu and Qin [23] presented three mean semiabsolute deviation models under the assumption that security returns are uncertain variables. Huang [24] proposed two new mean-variance and mean-semivariance models in which security returns are given subject to experts' estimations. Later, Huang [25] defined a new risk measurement, that is, a risk index, and further proposed a new mean-risk index selection method for portfolio selection based on the new risk measurement. Other portfolio selection methods based on uncertainty theory can be found in [26].

Artificial bee colony (ABC) algorithm is a fairly new metaheuristic proposed by Karaboga [27], which is based on simulating the foraging behavior of honey bee swarms. Based on some classic benchmark functions, the performance of the ABC algorithm was compared with that of some other population-based algorithms such as genetic algorithm (GA), differential evolution (DE), and particle swarm optimization (PSO) in [28–31]. Their research results demonstrated that the ABC algorithm is competitive to other population-based algorithms with an advantage of employing fewer control parameters. Recently, ABC algorithm has captured much attention and has been applied to many practical optimization problems including resource constrained project scheduling problem [32], reliability redundancy allocation problem [33], job shop scheduling problem [34], mechanical design problem [35], and traveling salesman problem [36]. A survey about applications of ABC algorithm is provided by Karaboga et al. in [37].

The purposes of this paper are to propose an uncertain portfolio adjusting model under the assumption that security returns are given mainly by experts' estimations, in which four criteria, namely, return, risk, transaction cost, and diversification degree of portfolio, are considered, and to develop a modified ABC algorithm for solving the proposed portfolio problem. The rest of the paper is organized as follows. For better understanding of the paper, some necessary knowledge about uncertain variable will be introduced in Section 2. In Section 3, we will propose an uncertain mean-variance-entropy model for portfolio selection. In Section 4, we develop a modified ABC algorithm to solve the proposed model. After that, an example is given to illustrate the proposed model and the effectiveness of the proposed algorithm in Section 5. Finally, some concluding remarks are given in Section 6.

## 2. Preliminary

Next, we will introduce some fundamental concepts and properties of uncertainty theory, which will be used throughout this paper.

**Definition 1 (Liu [17]).**

Let Γ be a nonempty set and let *L* be a *σ*-algebra over Γ. Each element Λ ∈ *L* is called an event. A set function *M*{Λ} is called an uncertain measure if it satisfies the following three axioms.

Axiom 1 (normality axiom): *M*{Γ} = 1.
Axiom 2 (duality axiom): *M*{Λ} + *M*{Λ^*c*^} = 1.
Axiom 3 (subadditivity axiom): for every countable sequence of events {Λ_*i*_}, we have
  (1) $$\begin{array}{c} M\left\{\overset{\infty }{\underset{i=1}{⋃}}\Lambda_{i}\right\}\leq \overset{\infty }{\underset{i=1}{\sum }}M\left\{\Lambda_{i}\right\}. \end{array}$$

The triplet (Γ, *L*, *M*) is called an uncertainty space. In order to obtain an uncertain measure of compound event, a product uncertain measure was defined by Liu [38], thus producing the fourth axiom of uncertainty theory as follows.

Axiom 4 (product axiom): let (Γ_*k*_, *L*_*k*_, *M*_*k*_) be uncertainty spaces for *k* = 1,2,…. Then the product uncertain measure *M* is an uncertain measure satisfying
  (2) $$\begin{array}{c} M\left\{\overset{\infty }{\underset{k=1}{\prod }}\Lambda_{k}\right\}=\overset{\infty }{\underset{k=1}{⋀}}M_{k}\left\{\Lambda_{k}\right\}. \end{array}$$

**Definition 2 (Liu [17]).**

An uncertain variable is a measurable function *ξ* from an uncertainty space (Γ, *L*, *M*) to the set of real numbers; that is, for any Borel set *B* of real numbers, the set

(3) $$\begin{array}{c} \left\{\xi \in B\right\}=\left\{\gamma \in \Gamma \;|\;\xi \left(\gamma \right)\in B\right\} \end{array}$$

is an event.

In order to describe an uncertain variable, a concept of uncertainty distribution is defined as follows.

**Definition 3 (Liu [17]).**

The uncertainty distribution of an uncertain variable *ξ* is defined by

(4) $$\begin{array}{c} \Phi \left(x\right)=M\left\{\xi \leq x\right\}, \end{array}$$

for any real number *x*.

Let *ξ* be an uncertain variable with continuous uncertainty distribution Φ. Then the inverse function Φ^−1^ is called the inverse uncertainty distribution of *ξ*.

**Theorem 4 (Liu [39]).**

Let *ξ*_1_, *ξ*_2_,…, *ξ*_*n*_ be independent uncertain variables with uncertainty distributions Φ_1_, Φ_2_,…, Φ_*n*_, respectively. If *f*(*x*_1_, *x*_2_,…, *x*_*n*_) is strictly increasing with respect to *x*_1_, *x*_2_,…, *x*_*m*_ and strictly decreasing with respect to *x*_*m*+1_, *x*_*m*+2_,…, *x*_*n*_, then *ξ* = *f*(*ξ*_1_, *ξ*_2_,…, *ξ*_*n*_) is an uncertain variable with an inverse uncertainty distribution:

(5) Ψ−1(α)=f(Φ1−1(α),…,Φm−1(α),Φm+1−1(1−α),…,Φn−1(1−α)).

Based on the uncertain measure, the definitions of the expected value and variance of an uncertain variable can be given as follows.

**Definition 5 (Liu [17]).**

Let *ξ* be an uncertain variable. Then the expected value of *ξ* is defined by

(6) $$\begin{array}{c} E\left[\xi \right]=\overset{+\infty }{\underset{0}{\int }}M\left\{\xi \geq x\right\}-\overset{0}{\underset{-\infty }{\int }}M\left\{\xi \leq x\right\} \end{array}$$

provided that at least one of the two integrals is finite.

As a useful representation of expected value, it has been proved by Liu [17] that

(7) $$\begin{array}{c} E\left[\xi \right]=\overset{1}{\underset{0}{\int }}\Psi^{-1}\left(\alpha \right)d\alpha , \end{array}$$

where Ψ^−1^ is the inverse uncertainty distribution of uncertain variable *ξ*.

**Definition 6 (Liu [17]).**

Let *ξ* be an uncertain variable with finite expected value *e*. Then the variance of *ξ* is defined by

(8) $$\begin{array}{c} V\left[\xi \right]=E\left[{\left(\xi -e\right)}^{2}\right]. \end{array}$$

Let *ξ* be an uncertain variable with expected value *e*. If we only know its uncertainty distribution Ψ, then the variance is

(9) V[ξ]=∫0+∞M{(ξ−e)2≥x}dx=∫0+∞M{(ξ≥e+x)∪(ξ≤e−x)}dx≤∫0+∞(M{ξ≥e+x}+M{ξ≤e−x})dx=∫0+∞(1−Ψ(e+x)+Ψ(e−x))dx=2∫0+∞x(1−Ψ(e+x)+Ψ(e−x))dx.

In this case, it is always assumed that the variance is

(10) $$\begin{array}{c} \;V\left[\xi \right]=2\overset{+\infty }{\underset{0}{\int }}x\left(1-\Psi \left(e+x\right)+\Psi \left(e-x\right)\right)dx. \end{array}$$

The expected value and variance of an uncertain variable satisfy the following properties.

**Theorem 7 (Liu [38]).**

Let *ξ* and *η* be independent uncertain variables with finite expected values. Then for any real numbers *a* and *b*, one has

(11) E[aξ+bη]=aE[ξ]+bE[η],V[aξ+b]=a2V[ξ].

## 3. Uncertain Portfolio Selection Model

Suppose that an investor starts with an existing portfolio and considers to reallocate his/her wealth among *n* securities. In order to describe the problem conveniently, we use the following notations.

 
*x*_*j*_: the proportion invested in security *j*,
 
*r*_*j*_: the return rate of security *j*,
 
*l*_*j*_: the lower bound constraint on security *j*,
 
*u*_*j*_: the upper bound constraint on security *j*,
 
*k*_*j*_: the constant rate of transaction cost for the security *j*,
 
*j* = 1,2,…, *n*.

Transaction cost is an important factor considered by investors in financial markets. Arnott and Wanger [40] suggested that ignoring transaction costs would lead to an inefficient portfolio, whereas Sadjadi et al. [41] showed that adding transaction costs would assist decision makers to better understand the behavior of an efficient frontier. Similar to the researches in [42] and others, we assume that the transaction cost is a V-shaped function of differences between a new portfolio **x** = (*x*_1_, *x*_2_,…, *x*_*n*_) and the existing portfolio **x** ^0^ = (*x*_1_^0^, *x*_2_^0^,…, *x*_*n*_^0^). That is to say, the transaction cost of *j*th security can be expressed as

(12) $$\begin{array}{c} c_{j}=k_{j}\left|x_{j}-x_{j}^{0}\right|,\;j=1,2,\ldots ,n. \end{array}$$

Hence, the total transaction cost of the shift from **x** ^0^ to **x** is *C*(**x**; **x** ^0^) = ∑_*j*=1_^*n*^ *k*_*j*_ | *x*_*j*_ − *x*_*j*_^0^|.

Furthermore, the net return on the portfolio after paying transaction costs is given by

(13) $$\begin{array}{c} \overset{n}{\underset{j=1}{\sum }}r_{j}x_{j}-\overset{n}{\underset{j=1}{\sum }}k_{j}\left|x_{j}-x_{j}^{0}\right|. \end{array}$$

For a new investor, it can be taken that *x*_*j*_^0^ = 0, *j* = 1,2,…, *n*.

Barak et al. [43] pointed out that very small weighting of an asset will have no distinct influence on the portfolio's return, but the administrative and monitoring costs will be increased. Similarly, very high weighting in any asset will cause investors to suffer from a larger risk. Thus, quantity constraints have to be included in the portfolio model. Specifically, a minimum *l*_*j*_ and a maximum *u*_*j*_ for each asset *j* are given, and we impose that either *x*_*j*_ = 0 or *l*_*j*_ ≤ *x*_*j*_ ≤ *u*_*j*_. In addition, one of the shortcomings of Markowitz's model is that the portfolios are often extremely concentrated on a few assets, which is a contradiction to the notion of diversification [44]. As a result, some researchers, such as Jana et al. [44], Fang et al. [45], Kapur [46], and Zhang et al. [47], have applied the entropy measure proposed by Shannon [48] to infer how much the portfolio is diversified. In this paper, we also employ entropy to measure the diversification degree of portfolio. That is to say, we maximize the entropy function:

(14) $$\begin{array}{c} S\left(x\right)=-\overset{n}{\underset{j=1}{\sum }}x_{j}\ln x_{j}. \end{array}$$

Assume that the objective of the investor wants to minimize the portfolio risk, and to maximize both the portfolio return after paying the transaction costs and the diversification degree of portfolio. Thus, the portfolio selection problem can be formulated as the following three-objective programming problem:

(15)  min⁡ V[∑j=1nrjxj−∑j=1nkj|xj−xj0|] max⁡ E[(∑j=1nrjxj)−∑j=1nkj|xj−xj0|] max⁡ −∑j=1nxjln⁡xj s.t. ∑j=1nxj=1, lj≤xj≤uj, j=1,2,…,n, xj≥0, j=1,2,…,n,

where *V* denotes the variance operators and *E* denotes the expected value operator.

There are many methods to solve the above multiobjective optimization problem (15). One basic method is to transfer the multiobjective optimization problem into a single-objective optimization problem. We can divide these methods into two different types. The first alternative is to construct only one evaluation function for optimization by weighting the multiple objective functions. Initial work on the weighted-sum method can be found in [49] with many subsequent applications and citations. Rao and Roy proposed a method for determining weights based on fuzzy set theory [50]. Marler and Arora provided insight into how the weighted-sum method works and have explored the significance of the weights with respect to preferences, the Pareto optimal set, and the objective-function values [51]. The second alternative is to select one important objective function as the objective function to optimize, while the rest of objective functions are defined as constrained conditions. For example, Marglin developed the *ɛ*-constraint method, where one individual objective function is minimized with an upper level constraint imposed on the other objective functions [52]. Ehrgott and Ruzika presented two modifications by first including slack variables in the formulation and second elasticizing the constraints and including surplus variables [53]. More researches for the multiobjective optimization methods can be found in [54–57]. In this paper, based on the second method, the problem (15) can be transformed into the following optimization problem:

(16)  min⁡ V[∑j=1nrjxj−∑j=1nkj|xj−xj0|] s.t. E[(∑j=1nrjxj)−∑j=1nkj|xj−xj0|]≥μ0, −∑j=1nxjln⁡⁡xj≥h0, ∑j=1nxj=1, lj≤xj≤uj, j=1,2,…,n, xj≥0, j=1,2,…,n,

where *μ*_0_ is a required rate of return of portfolio and *h*_0_ is the minimum entropy level the investors can tolerate.

In order to apply model (16) in a practical investment problem, we need to estimate security returns. The favored method is to regard security returns as random variables and then according to the historical observation take the arithmetic mean as the expected returns. However, since the security returns, especially short term security returns, are sensitive to various economic and noneconomic factors, it is found in reality that sometimes the historical data can hardly reflect the future security returns. In this situation, security returns have to be given mainly by experts' judgements and estimations rather than historical data. As is mentioned before, uncertainty theory provides a new tool to deal with subjective or empirical data. In this paper, we employ uncertain variables to describe the security returns. For the sake of simplicity, throughout this paper, the uncertain return *r*_*j*_ is a zigzag uncertain variable *r*_*j*_ ~ *Z*(*a*_*j*_, *b*_*j*_, *c*_*j*_), *j* = 1,2,…, *n*. *r*_*j*_ can be described with the following uncertainty distribution:

(17) $$\begin{array}{c} \Phi_{j}\left(r\right)=\{\begin{array}{cc} 0, & \text{if}\;\;r\leq a_{j},\\ \frac{\left(r-a\right)}{2\left(b-a\right)}, & \text{if}\;\;a_{j}\leq r\leq b_{j},\\ \frac{\left(r+c-2b\right)}{2\left(c-b\right)}, & \text{if}\;\;b_{j}\leq r\leq c_{j},\\ 1, & \text{if}\;\;r\geq c_{j}, \end{array} \end{array}$$

where *a*_*j*_ < *b*_*j*_ < *c*_*j*_.

According to Definitions 5 and 6, the expected value and the variance of the zigzag uncertain variable *ξ* ~ *Z*(*a*, *b*, *c*) are given as

(18) E[ξ]=a+2b+c4,V[ξ]=33α3+21α2β+11αβ2−β3384α,

where *α* = max⁡⁡{*b* − *a*, *c* − *b*} and *β* = min⁡⁡{*b* − *a*, *c* − *b*}.

**Theorem 8 (Liu [38]).**

Assume that *ξ*_1_ and *ξ*_2_ are independent zigzag uncertain variables *Z*(*a*_1_, *b*_1_, *c*_1_) and *Z*(*a*_2_, *b*_2_, *c*_2_), respectively. Then the sum *ξ*_1_ + *ξ*_2_ is also a zigzag uncertain variable *Z*(*a*_1_ + *a*_2_, *b*_1_ + *b*_2_, *c*_1_ + *c*_2_); that is,

(19) $$\begin{array}{c} Z\left(a_{1},b_{1},c_{1}\right)+Z\left(a_{2},b_{2},c_{2}\right)=Z\left(a_{1}+a_{2},b_{1}+b_{2},c_{1}+c_{2}\right). \end{array}$$

The product of a zigzag uncertain variable *Z*(*a*, *b*, *c*) and a scalar number *k* > 0 is also a zigzag uncertain variable *Z*(*ka*, *kb*, *kc*); that is,

(20) $$\begin{array}{c} k\cdot Z\left(a,b,c\right)=Z\left(ka,kb,kc\right). \end{array}$$

Therefore, for any real numbers *x*_*j*_ ≥ 0, *j* = 1,2,…, *n*, the total uncertain return is still a zigzag uncertain variable in the form of

(21) rp=∑j=1nrjxj~Z(∑j=1najxj,∑j=1nbjxj,∑j=1ncjxj).

Furthermore, according to Theorem 7, the net uncertain expected return of the portfolio after paying transaction costs is given as

(22) E[rp−C(x)]=E[∑j=1nrjxj−∑j=1nkj|xj−xj0|]=E[∑j=1nrjxj]−∑j=1nkj|xj−xj0|=14∑j=1n(aj+2bj+cj)xj−∑j=1nkj|xj−xj0|,

and the uncertain variance of the portfolio after paying transaction costs is

(23) V[rp−C(x)]=V[∑j=1nrjxj−∑j=1nkj|xj−xj0|]=V[∑j=1nrjxj]=33M3+21M2m+11Mm2−m3384M=11M2128+7Mm128+11m2384−m3384M,

where

(24) $$\begin{array}{c} M=\max\left\{\overset{n}{\underset{j=1}{\sum }}\left(b_{j}-a_{j}\right)x_{j},\overset{n}{\underset{j=1}{\sum }}\left(c_{j}-b_{j}\right)x_{j}\right\},\\ m=\min\left\{\overset{n}{\underset{j=1}{\sum }}\left(b_{j}-a_{j}\right)x_{j},\overset{n}{\underset{j=1}{\sum }}\left(c_{j}-b_{j}\right)x_{j}\right\}. \end{array}$$

To simplify the portfolio selection model, it seems reasonable to choose the securities in such a way that *b*_*j*_ − *a*_*j*_ ≤ *c*_*j*_ − *b*_*j*_,  *j* = 1,2,…, *n*. Therefore, model (16) can be converted into the following crisp form:

(25)  min⁡ 11128(∑j=1n(cj−bj)xj)2+7128(∑j=1n(cj−bj)xj) ×(∑j=1n(bj−aj)xj)+11384(∑j=1n(bj−aj)xj)2 −(∑j=1n(bj−aj)xj)3384(∑j=1n(cj−bj)xj) s.t. 14∑j=1n(aj+2bj+cj)xj−∑j=1nkj|xj−xj0|≥μ0, −∑j=1nxjln⁡⁡xj≥h0, ∑j=1nxj=1, lj≤xj≤uj ,j=1,2,…,n, xj≥0, j=1,2,…,n.

It should be noted that the proposed uncertain portfolio model (25) is different from the Markowitz M-V model because it is based on the different theories. That is to say, if the security returns are characterized as random variables with probability distributions, we can study portfolio selection problems by probability theory, while if security returns are regarded as uncertain variables with uncertainty distributions, we can apply uncertainty theory to do researches on the relevant problems.

## 4. Modified ABC Algorithm

### 4.1. Standard ABC Algorithm

Artificial bee colony (ABC) algorithm is a new swarm intelligence method which simulates intelligent foraging behavior of honey bees and it is initially proposed by Karaboga [27]. In the ABC algorithm, there are three types of bees: the employed bee, the onlooker bee, and the scout bee. Each of them plays different roles in the process: the employed bees stay on a food source and provide the neighborhood of the source in its memory; the onlooker bee gets the information of food sources from the employed bees in the hive and selects one of the food sources to gather the nectar; and the scout bee is responsible for finding a new food source.

In each successful algorithm, a robust search process, including exploitation and exploration process, must be implemented effectively. In the ABC algorithm, the employed bees and onlookers execute the exploitation process in the search space, while the scout bees execute the exploration process. There is only one employed bee around each food source. In other words, the number of employed bees is equal to the number of food sources around the hive. The positions of food sources represent possible solutions to the optimization problem and the nectar amount of a food source corresponds to the quality or fitness of the associated solution. The number of the employed bees or the onlooker bees is equal to the number of solutions in the population.

At the first step, the ABC generates a randomly distributed initial population *P* of *SN* solutions (food source positions), where *SN* denotes the size of population. Each solution *X*_*i*_  (*i* = 1,2,…, *SN*) is a *D*-dimensional vector. Here, *D* is the number of optimization parameters. After initialization, the population is subjected to repeated cycles of four major steps: updating feasible solutions by employed bees, selection of feasible solutions by onlooker bees, updating feasible solutions by onlooker bees, and avoidance of suboptimal solutions by scout bees.

*Updating Feasible Solutions by Employed Bees*. In order to produce a new feasible food source from the old one in memory, the ABC uses the following expression:

(26) $$\begin{array}{c} v_{ij}=x_{ij}+\phi_{ij}\left(x_{ij}-x_{kj}\right), \end{array}$$

where *k* ∈ 1,2,…, *SN* and *j* ∈ 1,2,…, *D* are randomly chosen indexes. Although *k* is determined randomly, it has to be different from *i*. *ϕ*_*ij*_ is a random number in [−1,1].

If a new food source *V*_*i*_ is better than an old food source *X*_*i*_, the old food source is replaced by the new food source. Otherwise, the old one is retained.

*Selection of Feasible Solutions by Onlooker Bees*. After all employed bees complete the search process, they share the information about their food sources with onlooker bees. An onlooker bee evaluates the nectar information obtained from all employed bees and chooses a food source depending on the probability value *p*_*i*_ associated with that food source:

(27) $$\begin{array}{c} p_{i}=\frac{\text{fi}{\text{t}}_{i}}{\sum_{n=1}^{SN}\text{fi}{\text{t}}_{n}}, \end{array}$$

where fit_*i*_ is the fitness value of the solution *i* which is proportional to nectar amount of the food source in the position *i*.

*Updating Feasible Solutions by Onlooker Bees*. Based on the information obtained from the employed bees, the onlooker bees select their feasible food sources. The selected food sources are then updated using (26); that is, an old food source is replaced by a new food source if the new food source is of a better quality.

*Avoidance of Suboptimal Solutions by Scout Bees*. If a position cannot be improved further through a predetermined number of cycles called limit, then employed bees will abandon the food source positions and become scout bees. This helps avoid suboptimal solutions. Assume that the abandoned source is *X*_*i*_ and *j* ∈ 1,2,…, *D*; then the scout discovers a new food source by using the equation below:

(28) $$\begin{array}{c} x_{i,j}=x_{\min}^{j}+\text{rand}\left(0,1\right)\left(x_{\max}^{j}-x_{\min}^{j}\right), \end{array}$$

where *j* is determined randomly; it should be noticed that it has to be different from *i*.

The main steps of the standard ABC algorithm are given in Algorithm 1.

### 4.2. Modified Artificial Bee Colony Algorithm

#### 4.2.1. Chaotic Initialization

Generally speaking, diversity in initial population helps escape from local optima and good-quality initial solutions accelerate convergence speed in an evolutionary algorithm. If no information about the solution is available, then random initialization is the most commonly used method to initialize the population. However, this method may affect the algorithm performance on the convergence speed and the quality of the final solution. Recently, chaotic maps with ergodicity, irregularity, and the stochastic property have been adopted to initialize the population, and some good results have been shown in many applications [58, 59]. In this paper, chaos-based initialization method is employed to increase the population diversity and improve the global convergence.

A simplest chaotic map is logistic map, whose equation is the following:

(29) $$\begin{array}{c} x_{n+1}=\mu x_{n}\left(1-x_{n}\right), \end{array}$$

where *μ* is a control parameter and *x*_*n*_ is the *n*th chaotic number, where *n* denotes the iteration number. Obviously, *x*_*n*_ ∈ (0,1) under the conditions that the initial *x*_0_ ∈ (0,1). In particular, *x*_*n*_ behaves as chaotic dynamics when *μ* = 4 and *x*_0_ ∉ {0,0.25,0.5,0.75,1}.

The pseudocode of the proposed chaotic initialization is given in Algorithm 2.

#### 4.2.2. New Search Mechanism

The employed bee and onlooker bee produce a new feasible food source according to (26). However, the convergence performance of the algorithm is not good in some cases. Therefore, we proposed a modified search method to produce new solutions, in which forgetting factor and neighborhood factor are considered. This operation can be defined as follows:

(30) $$\begin{array}{c} v_{i,j}=\varphi x_{i,j}+\psi \phi_{ij}\left(x_{i,j}-x_{k,j}\right), \end{array}$$

where *φ* is the forgetting factor, which expresses the memory strength for current food source, and it is decreased gradually. In addition, the quality of the neighborhood food source *X*_*k*_ also affects the convergence speed and quality of the final solution. Thus, the neighborhood factor *ψ* is introduced to accelerate the convergence speed by adjusting the radius of the search for new candidates. The *φ* and *ψ* are defined as follows:

(31) $$\begin{array}{c} \varphi =\omega_{\max}-\frac{\text{iteration}}{\text{MCN}}\left(\omega_{\max}-\omega_{\min}\right),\\ \psi =\omega_{\min}+\frac{\text{iteration}}{\text{MCN}}\left(\omega_{\max}-\omega_{\min}\right), \end{array}$$

where the values of *ω*_max⁡_ and *ω*_min⁡_ represent the maximum and minimum percentage of the position adjustment for the employed bees or onlooker bees. With these selected values, the value of *φ* will linearly decrease and the value of *ψ* will linearly increase.

Moreover, in the scout bee phase, if the food source is abandoned, then the scouts produce a new food source by (28). However, to some extent, it has the defects of prematurity and stagnation. Therefore, the chaotic search technique is used to get out of the local optima and is specifically illustrated in Algorithm 3.

#### 4.2.3. The Proposed Method

Based on the above discussions, the modified ABC algorithm can be well balanced between the exploration and exploitation. The pseudocode of the proposed algorithm is given in Algorithm 4.

## 5. Numerical Example

In this section, we give a numerical example to illustrate the application of the uncertain mean-variance-entropy model with transaction costs and the effectiveness of the proposed algorithm. In the example, security returns cannot be well reflected by the historical data and are given by experts' evaluations. The investor wants to select portfolios from 8 individual securities. Thus, we assume that the return of security *j* is a zigzag uncertain variable *r*_*j*_ ~ *Z*(*a*_*j*_, *b*_*j*_, *c*_*j*_), *j* = 1,2,…, 8. The uncertainty distributions of security returns are given in Table 1. And other related portfolio parameters are also listed in Table 1.

To solve the proposed model (25) by the modified ABC algorithm, we let the number of food sources *SN* = 20, the dimension of the problem *D* = 8, maximum cycle number MCN = 1000, and the control parameter limit = 100. Moreover, in this section, a total of 10 runs for each experimental setting are conducted and the average results are given. The values of *ω*_max⁡_ and *ω*_min⁡_ are fixed to 1 and 0.2, respectively.

Suppose that the minimum entropy level *h*_0_ = 1.8 and the efficient portfolios under different expected returns are shown in Table 2. If the investor is not satisfied with any of the portfolios obtained, more portfolios can be obtained by varying the value of *μ*. From Table 2, we can see that the larger the return of portfolio is, the larger the risk of portfolio is. There is a tradeoff between the return and the risk. Moreover, for the different expected return *μ*, the optimal portfolios are also different.

Next, to demonstrate that the transaction costs have effect on the optimal portfolio selection, given *h*_0_ = 1.7 and *μ* = 0.08, the efficient portfolios under different transaction costs are given in Table 3. It should be noted that, for simplicity, we only vary the transaction cost of security 5. We can see that although the proportions of securities 2, 4, and 6–8 are the same, the holdings of other securities are different. Simultaneously, we also see that the total transaction cost also increases.

**Figure 1:**
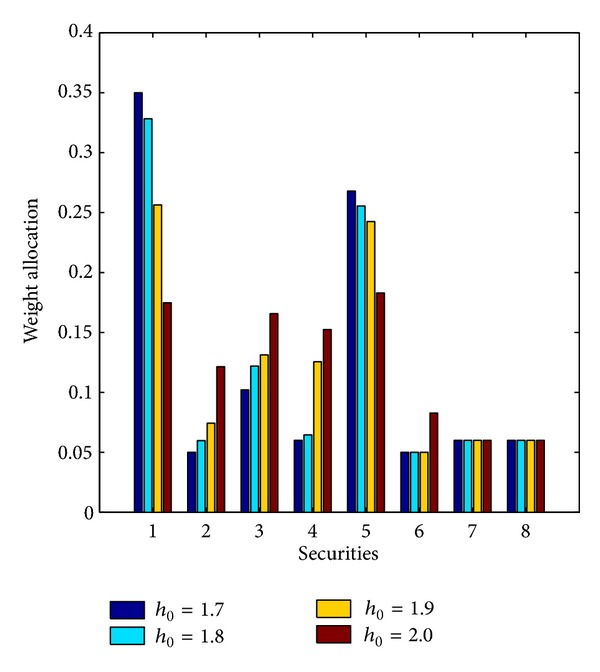
Comparison results under different diversification degrees.

Figure 1 gives comparison results of the optimal portfolio strategies under different diversification degrees; that is, *h*_0_ = 1.7, *h*_0_ = 1.8, *h*_0_ = 1.9, and *h*_0_ = 2.0. The result indicates that, as the diversification degree of the portfolio changes, the investors adopt different investment strategies.

**Figure 2:**
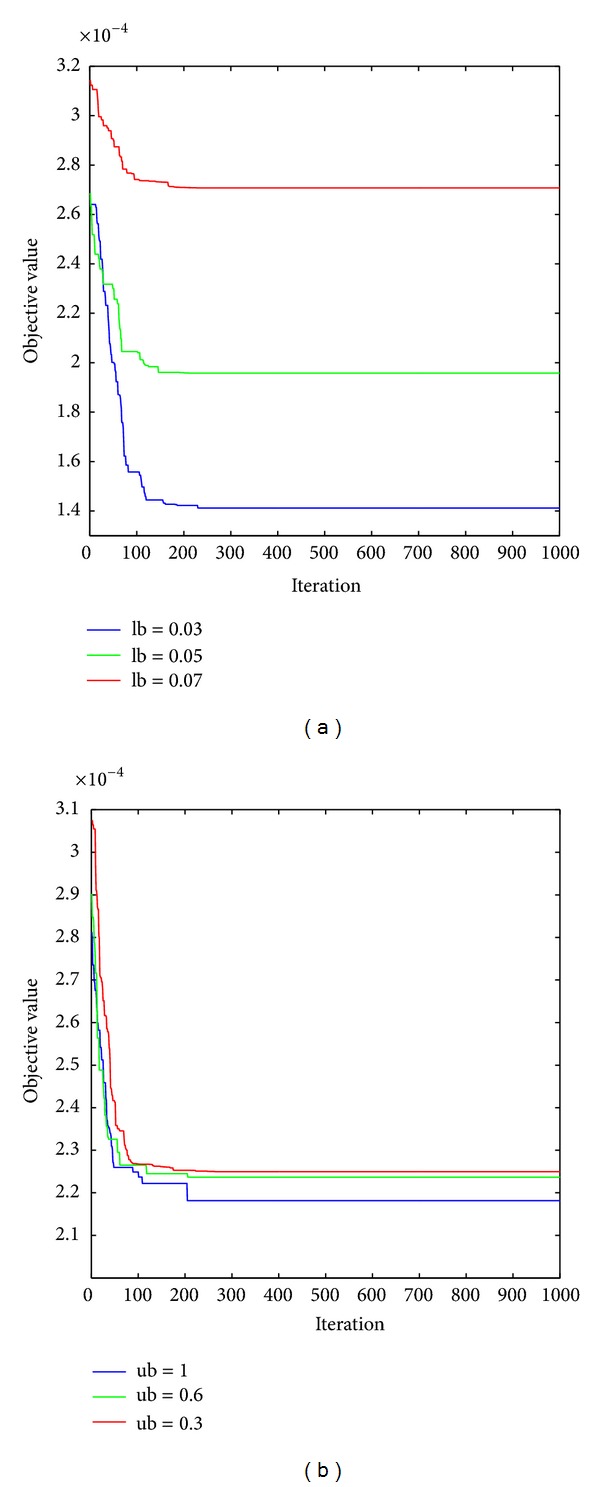
The performance of the ABC algorithm under different bound constraints.

Figure 2 presents the performance of the ABC algorithm with respect to the choice of the lower and upper bounds. Moreover, to show the robustness of the proposed algorithm, we use relative error as the index; that is, (maximum − actual  value)/maximum × 100%, where the maximum is the maximal value of all the objective values calculated. The detailed results are shown in Table 4. Obviously, the relative errors do not exceed 1%. That is, the proposed algorithm is robust to set parameters.

**Figure 3:**
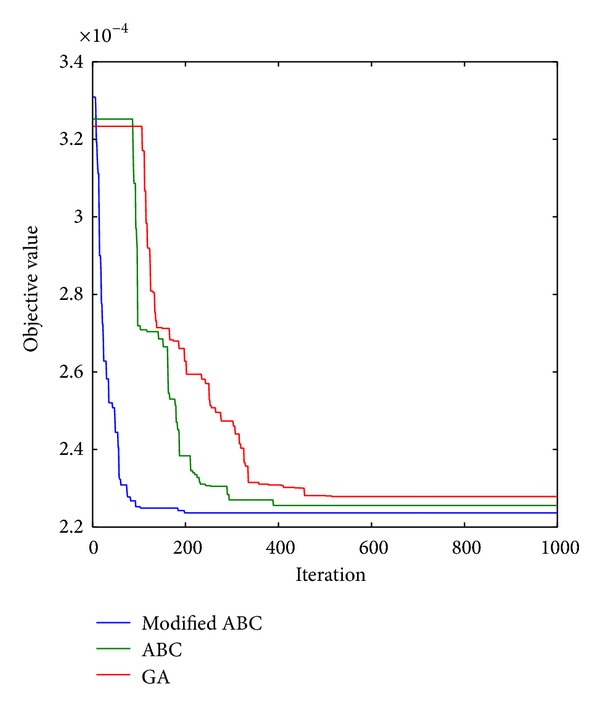
Convergence characteristics of the modified ABC, ABC, and GA (*u*_0_ = 0.08, *h*_0_ = 1.8).

Table 5 presents a comparison of the modified ABC, ABC, and GA. From Table 5, it can be observed that, for the *u*_0_ = 0.09 and *h*_0_ = 1.75, the investment strategy obtained by modified ABC algorithm outperforms those produced by standard ABC algorithm and GA, because the global optimal value obtained by modified ABC algorithm is minimal. In addition, Figure 3 shows the convergence characteristic of modified ABC, ABC, and GA. Generally speaking, the modified ABC algorithm converges within 200 iterations, while ABC and GA algorithms converge within 400 and 430 iterations, respectively. All these results show that the modified ABC algorithm has a better performance and is efficient in finding optimal portfolios.

## 6. Conclusion

Since the security market is so complex, sometimes security returns have to be predicted mainly by experts' evaluations rather than historical data. This paper discusses a portfolio selection problem with returns given by experts' evaluations. By taking the security returns as uncertain variables, this paper makes use of the uncertain expected value and uncertain variance to measure the return and risk of securities. Furthermore, we propose an uncertain mean-variance-entropy model for portfolio selection with transaction costs. After that, a modified ABC algorithm is developed to solve the proposed problem. Finally, a numerical example is presented to illustrate this modeling concept and to demonstrate the effectiveness of the proposed algorithm. The results show that the proposed method is applicable and effective for solving the portfolio selection problem.

## Figures and Tables

**Algorithm 1:**
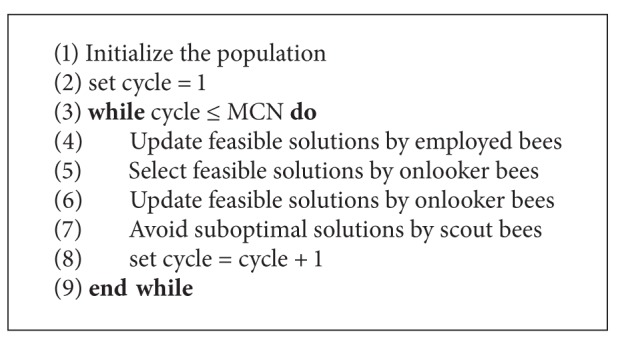
The framework of the standard ABC algorithm.

**Algorithm 2:**
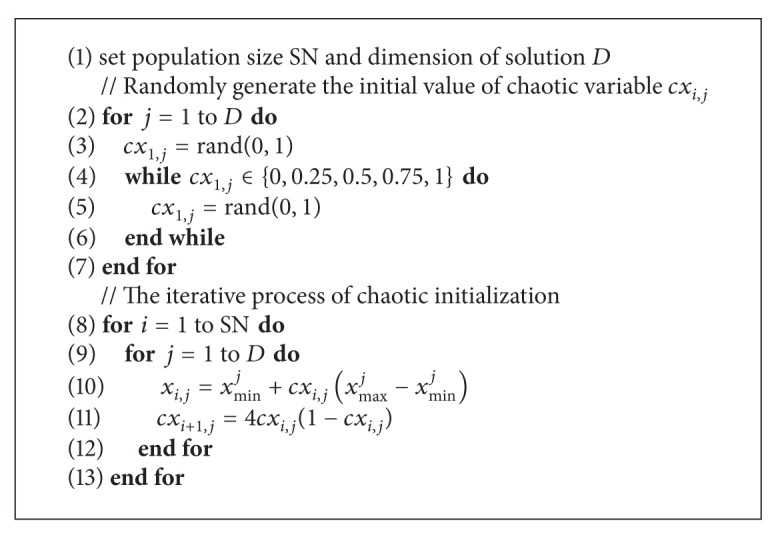
Chaotic initialization.

**Algorithm 3:**
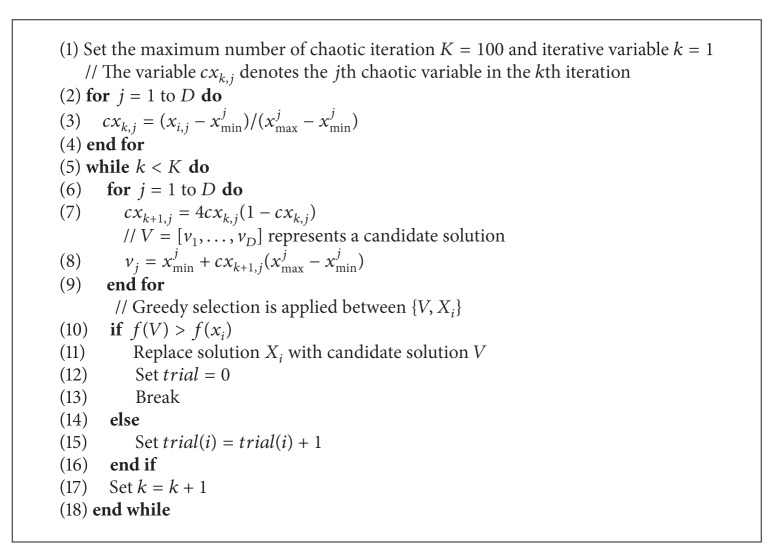
Chaotic search for scout bees.

**Algorithm 4:**
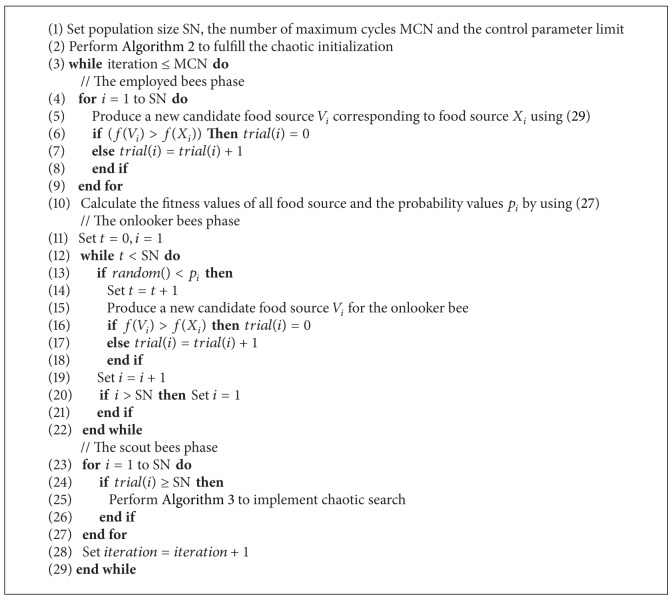
Modified ABC algorithm.

**Table 1:** The zigzag uncertain returns of 8 securities.

Security	1	2	3	4	5	6	7	8
*a*_*j*_	0.006	−0.017	0.085	0.050	0.163	−0.035	0.095	0.116
*b*_*j*_	0.011	−0.035	0.103	0.062	0.172	−0.082	0.161	0.235
*c*_*j*_	0.028	0.013	0.128	0.095	0.193	0.022	0.232	0.527
*x*_*j*_^0^	0.05	0.07	0.15	0.06	0.15	0.22	0.13	0.17
*k*_*j*_	0.002	0.005	0.003	0.001	0.004	0.002	0.005	0.003
*l*_*j*_	0.1	0.05	0.09	0.06	0.07	0.05	0.06	0.06
*u*_*j*_	0.35	0.35	0.35	0.35	0.35	0.35	0.35	0.35

**Table 2:** The efficient portfolios with *h*_0_ = 1.8.

Security	*x*_1_	*x*_2_	*x*_3_	*x*_4_	*x*_5_	*x*_6_	*x*_7_	*x*_8_	Risk
*μ*_0_ = 0.07	0.349	0.050	0.137	0.072	0.222	0.050	0.060	0.060	0.0002236
*μ*_0_ = 0.09	0.309	0.050	0.090	0.098	0.283	0.050	0.060	0.060	0.0002255
*μ*_0_ = 0.11	0.136	0.063	0.212	0.068	0.350	0.050	0.060	0.060	0.0002406

**Table 3:** The efficient portfolios under different transaction costs (*μ*_0_ = 0.08, *h*_0_ = 1.7).

Security	*x*_1_	*x*_2_	*x*_3_	*x*_4_	*x*_5_	*x*_6_	*x*_7_	*x*_8_	TC
*k*_5_ = 0	0.341	0.050	0.090	0.060	0.289	0.050	0.060	0.060	0.00188
*k*_5_ = 0.004	0.350	0.050	0.102	0.060	0.268	0.050	0.060	0.060	0.00234
*k*_5_ = 0.008	0.332	0.050	0.096	0.060	0.292	0.050	0.060	0.060	0.00298
*k*_5_ = 0.015	0.322	0.050	0.105	0.060	0.293	0.050	0.060	0.060	0.00394

**Table 4:** Robustness analysis for the modified ABC algorithm (*u*_0_ = 0.08, *h*_0_ = 1.8).

SN	MCN	Limit	Objective value	Relative error (%)
10	1500	50	2.2474*E* − 4	0
20	2000	100	2.2445*E* − 4	0.13
20	1500	150	2.2424*E* − 4	0.22
30	2500	150	2.2469*E* − 4	0.02
30	2000	100	2.2369*E* − 4	0.47
40	3000	200	2.2384*E* − 4	0.4
40	1500	100	2.2406*E* − 4	0.3
50	1500	100	2.2372*E* − 4	0.45
60	2000	150	2.2371*E* − 4	0.46
70	2000	50	2.2358*E* − 4	0.52

**Table 5:** The comparison of the modified ABC, ABC, and GA (*u*_0_ = 0.09, *h*_0_ = 1.75).

	Modified ABC	ABC	GA
*x*_1_	0.327	0.319	0.311
*x*_2_	0.050	0.050	0.061
*x*_3_	0.090	0.132	0.158
*x*_4_	0.060	0.060	0.060
*x*_5_	0.303	0.269	0.240
*x*_6_	0.050	0.050	0.050
*x*_7_	0.060	0.060	0.060
*x*_8_	0.060	0.060	0.060
Risk	2.203*E* − 4	2.234*E* − 4	2.270*E* − 4
